# Laterality in Emotional Language Processing in First and Second Language

**DOI:** 10.3389/fpsyg.2021.736359

**Published:** 2022-02-03

**Authors:** Raheleh Heyrani, Vahid Nejati, Sara Abbasi, Gesa Hartwigsen

**Affiliations:** ^1^Department of Education and Psychology, Alzahra University, Tehran, Iran; ^2^Raftar Cognitive Neuroscience Research Center, Shahid Beheshti University, Tehran, Iran; ^3^Department of Education and Psychology, Shahid Beheshti University, Tehran, Iran; ^4^Institute for Cognitive Science Studies, Tehran, Iran; ^5^Lise Meitner Research Group Cognition and Plasticity, Max Planck Institute for Human Cognitive and Brain Sciences, Leipzig, Germany

**Keywords:** bilingualism, emotional words, foreign language, lateralization, left hemisphere, mother tongue language, right hemisphere

## Abstract

Language is a cognitive function that is asymmetrically distributed across both hemispheres, with left dominance for most linguistic operations. One key question of interest in cognitive neuroscience studies is related to the contribution of both hemispheres in bilingualism. Previous work shows a difference of both hemispheres for auditory processing of emotional and non-emotional words in bilinguals and monolinguals. In this study, we examined the differences between both hemispheres in the processing of emotional and non-emotional words of mother tongue language and foreign language. Sixty university students with Persian mother tongue and English as their second language were included. Differences between hemispheres were compared using the dichotic listening test. We tested the effect of hemisphere, language and emotion and their interaction. The right ear (associated with the left hemisphere) showed an advantage for the processing of all words in the first language, and positive words in the second language. Overall, our findings support previous studies reporting left-hemispheric dominance in late bilinguals for processing auditory stimuli.

## Introduction

Language is a cognitive function that is asymmetrically distributed across both hemispheres. Current models on the functional neuroanatomy of language favor left hemispheric dominance for key linguistic operations in language comprehension and production (e.g., Hickok and Poeppel, 2007; Friederici, 2011; Friederici and Gierhan, 2013; Hagoort and Indefrey, 2014). These models argue that language functions result from the interactions of distant temporal, frontal, and parietal brain regions within a left-lateralized network. In particular, the dual-stream model of language (Hickok and Poeppel, 2007; Rauschecker and Scott, 2009) suggests that a ventral stream maps sound onto meaning by connecting bilateral middle temporal areas with the left ventrolateral prefrontal cortex. In contrast, a dorsal, left-lateralized stream maps are involved in articulation via the interaction of superior temporal lobe, and premotor as well as prefrontal cortex. These streams are underpinned by distinct anatomical fiber tracts that connect temporal areas with frontal regions (e.g., Friederici and Gierhan, 2013).

Lateralization of language function has been demonstrated both in patients with brain lesions and in healthy volunteers in numerous studies since the early observations by Broca and Wernicke (e.g., Price, 2000; Szaflarski et al., 2002). Many studies investigated hemispheric asymmetries by means of behavioral measures, including response accuracy, reaction time, and laterality indices. These studies demonstrated an advantage of left-hemispheric lateralization for language comprehension and right-hemispheric lateralization for spatial attention in right-handers relative to left-handed participants (Pujol et al., 1999; Price, 2000; Wilkinson and Halligan, 2002; Vogel et al., 2003; Powell et al., 2012; Prieur et al., 2017). Typical lateralization is most predominant for both right-handers and left-handers, but atypical lateralization is more common in left-handers. Furthermore, left-handers demonstrated a more variable distribution across both hemispheres, supporting a less focal profile of functional brain lateralization. Together, these findings emphasize the role of individual differences in brain asymmetries and related cerebral dominance mechanisms (O’Regan and Serrien, 2018).

Left-hemispheric language areas include the left posterior superior temporal gyrus, portions of the left anterior temporal lobe, the inferior parietal lobe, the left inferior frontal cortex, and the left insular cortex. In general, these brain areas and their functional and structural connections represent the basis of the left perisylvian language network (Hagoort, 2013). However, despite a left-hemispheric dominance, the right hemisphere also contributes to language (Pujol et al., 1999). For instance, several right-hemispheric regions, including the inferior frontal gyrus and premotor cortex as well as posterior temporal gyrus and sulcus play a role in prosody processing (Sammler et al., 2015). In summary, while previous work strongly argues for a left-hemispheric dominance of language, the right hemisphere also makes a substantial contribution to language function.

The ability to use two languages equally well is referred to as bilingualism (Basnight-Brown, 2014). Due to the challenge of managing two languages in bilinguals, the neural underpinnings in the perception of words, thoughts and communication structure may be different from monolinguals (Freeman et al., 2016). There are controversial hypotheses about the role of each hemisphere in language processing in the bilingual brain. Based on the comparison of monolinguals and bilinguals, it was argued that both monolinguals and bilinguals who learn a second language at later age show left hemispheric dominance. In contrast, early bilingualism was associated with significant activity in both hemispheres (Hull and Vaid, 2006). A neuroimaging study that included word production in different languages showed similar neural activity patterns for four languages across participants (Briellmann et al., 2004). While language lateralization was not formally assessed in this study, multi-linguals who learned the second language late, showed stronger activity in the left hemisphere.

Contrary to the critical contribution of left perisylvian regions to specific linguistic operations outlined above, the neural networks supporting pragmatic aspects of verbal communication in native and non-native languages (L1 and L2, respectively) have mainly been linked with the right hemisphere (RH) (Calvo et al., 2019). Accordingly, emotional prosody usually shows strong lateralization to the right hemisphere (e.g., Heilman et al., 1984; Hoekert et al., 2010; see Hartwigsen and Siebner, 2012). Nevertheless, several reports have shown that left-hemisphere activity is also associated with pragmatic domains (e.g., prosody, indirect speech, and figurative language), with the strength of the involvement being similar or even greater than that observed in the RH (Kreitewolf et al., 2014). For instance, lateralization of prosody processing seems to depend on its linguistic function (e.g., Belyk and Brown, 2014; van der Burght et al., 2019; for a meta-analysis) For instance, van der Burght et al. (2019) found increased activity in the left prefrontal cortex when prosodic cues guided sentence comprehension. However, when prosodic cues were superfluous for establishing the sentence structure, activity was lateralized to the right prefrontal cortex. This observation shows that hemispheric asymmetries in the processing of meaningful stimuli strongly depend on the amount of linguistic information and its relevance for guiding language comprehension. These findings challenge the simplistic notion that pragmatic aspects of verbal communication are mainly processed in the right hemisphere. Accordingly, a case report on an adult bilingual patient showed preservation of pragmatic verbal skills in both languages (L1: Spanish, L2: English) despite bilateral damage that was stronger in right-hemispheric regions (Calvo et al., 2019). This study suggested that multiple functions of verbal communication can be spared despite extensive damage to the RH. Consequently, claims for a putative relation between pragmatics and the RH may have been overemphasized in the monolingual and bilingual literature.

Humans communicate intentions to others by using (emotional) language. In particular, emotional words may change the way of interpersonal communication (Godfrey and Grimshaw, 2015). Two main factors are linked to the processing of emotional words: valence and arousal. The valence of a word varies from negative to positive and is defined as a measure of how pleasant or unpleasant a word is, whereas arousal ranges from calm to highly arousing and is defined as a measure of how intensely a person would want to approach or avoid something (Kuperman et al., 2014). Previous work showed that the processing of emotional words differs from neutral words (Winskel, 2013). Specifically, the left hemisphere (LH) is more efficient in (neutral) language processing, while the RH is often linked to the processing of emotions (Abbassi et al., 2011). A recent study explored how emotional prosody modulates hemispheric asymmetry in language processing (Godfrey and Grimshaw, 2015). Employing a dichotic listening task, that study revealed a robust REA (Right Ear Advantage) when words were presented in neutral prosody, which was diminished in intensity for emotional prosody. However, the valence was not significantly influencing this effect, pointing toward similar effects for different emotions. These results support the notion that the right hemisphere is in charge of processing emotional prosody. Other studies revealed that the emotional content of words has a stronger effect when presented in a bilingual’s first language (L1) in comparison with their second language (L2) (Ferré et al., 2010; Conrad et al., 2011; Opitz and Degner, 2012; Chen et al., 2015; Kazanas and Altarriba, 2016; Rosselli et al., 2017). This is most likely explained by automatic processing of emotional words in L1 compared to slower, semantic processing in L2 (Ong et al., 2017). However, one study on Chinese-English bilinguals indicated that emotional word processing might be affected by the proficiency and complexity of the respective language (Ong et al., 2017). According to the Right Hemisphere Hypothesis (Killgore and Yurgelun-Todd, 2007), automatic processing is linked to the left hemisphere, while controlled processing engages the right hemisphere. A common assumption is that the right hemisphere plays a dominant role in emotional processing, with a key contribution of the amygdala. Emotional regulation and control of emotional thoughts and words, on the other hand, requires the contribution of key areas for cognitive control, attention and self-regulation in the anterior and posterior cingulate cortex (Ochsner and Gross, 2005). Yet, the specific contribution of both hemispheres to the processing of emotional words in L1 and L2 remains unclear to date. To address this issue, the present study examined hemispheric differences in the processing of (non) emotional words in the mother tongue and second language. To this end, we employed the dichotic listening test to compare the efficiency of both hemispheres in auditory perception of emotional and non-emotional words in L1 and L2.

## Materials and Methods

### Participants

A total of bilingual students (30 females) with Persian as mother tongue language (L1) and English as second language (L2) participated in this cross-sectional study. Age range was 18 to 44 (mean ± SD, 26.48 ± 5.67). Mean education was 17.07 ± 1.51 years. The following inclusion criteria were used: (i) mother tongue language had to be Farsi, second language had to be English, (ii) education in L2 started after the sixth year of life, (iii) no verbal, visual or auditory dysfunctions, (iv) right handedness using Edinburgh Handedness Inventory (EHI), and (v) at least an upper intermediate level in all of four English skills. The Laterality Quotient value was used to assess handedness. Accordingly, a quotient of less than −40 defined left handedness, values between −40 and +40 ambidexterity and values above +40 defined right handedness. We did not include participants with a known history of psychiatric or neurological disorders or current use of psychiatric medication. All participants had normal or corrected to normal vision. The English skills included listening, speaking, reading and writing. We defined bilingualism as the use of another language different from the mother tongue language (Hakuta, 2009). In our study, the second language was English. Language experience was assessed with a questionnaire that included questions about the participant’s first and second languages, such as the following examples: “At what age did you start learning English? How many percent of your daily conversations is in English? How did you learn the English language? What is your English test score and in which type of test? If you consider a native person’s level of English from 1 to 10, how do you rate your-self. For the selection of Persian words, we used free association in a survey. We then assessed these words according to previous studies: words as suggested by Namatzadeh (2016). We selected those negative and positive words that were included in a previous thesis from our group (Oruji, 2011). All selected negative, positive and neutral words were also used in Aliloo (2000), Farhangi (2003). Then, we matched the required word pairs for the number of similar letters. Each of the selected words was evaluated based on SAM in two levels of arousal and valence. The final set was piloted in a separate mini-study. For the selection of English words, 1031 words were chosen from a previous study (Bradley and Lang, 1999). These words were presented to three English teachers. Stimuli were rated on a Lickert scale ranging from 1 to 10 for frequency according to tutorial books used in their classes. Evaluations were summed and those words with high and close frequencies were chosen. These words were finally assessed with the SAM scale.

### Materials

The dichotic listening test was used in our study. As a prerequisite, we performed a survey study with 100 University students who did not participate in the main experiment. In the survey, participants were asked to write down as many positive, negative and neutral words as possible in half an hour. In total, we collected 5000 words including 1562 negative, 1525 positive, and 1413 neutral words. After screening, 133 negative, 93 positive, and 100 neutral words were extracted. Comparisons with other previous studies, and matching the number of letters in Persian and English words led to a final list of 24 positive, 24 negative, and 24 neutral words, including 12 positive, 12 negative, and 12 neutral words in Farsi (L1) and English (L2), respectively. We included 18 trials with pairs of 1 stimuli, which sums up to a total of 36 trials for each the task (dichotic listening test). Number of words was 72. Three types of emotions were included (positive, negative, and neutral), but not all of them were paired with each other. The full stimulus list is included in the Supplementary Material. With respect to the number of words per emotion, there was a final list of 24 positive, 24 negative, and 24 neutral words, including 12 positive, 12 negative, and 12 neutral words in Farsi (L1) and English (L2), respectively. An additional group of 60 University students rated all words on the self-assessment manikin (SAM) to determine the level of arousal and valence of each word and match this between word pairs in both languages for both tests (Morris, 1994; Bynion and Feldner, 2020). Accordingly, each word was rated in terms of arousal (low vs. high) and emotional valence (negative vs. positive). Each participant had 14 seconds to evaluate each word (7 seconds for the assessment of arousal and valence, respectively). Means and standard deviations of each word can be found in the Supplementary Material.

The dichotic listening test (Broadbent, 1954; Musiek and Chemerk, 2015) was used to study hemispheric asymmetry. Dichotic listening refers to different acoustic events presented to each ear simultaneously. Most commonly, the acoustic signals are speech, such as digits, words, or consonant-vowels or sentences. The dichotic listening test is the most frequently used method to reveal hemispheric dominance for language processing, particularly in the extraction of the phonetic code from the speech signal (Broadbent, 1954). It represents a measure of both temporal and frontal lobe function, attention and information processing speed and can be used to measure hemispheric asymmetries (see also Kimura, 1967). By recording the pattern of verbal responses to dichotic presentations of simple speech sounds, it is possible to infer the hemisphere in which receptive capabilities are most likely localized in each individual subject. In our study, word pairs were presented to both ears simultaneously and the participants had to say which words they heard, with their response being recorded. Individual scores per participant and ear were based on the number of produced words per condition (positive, negative, neutral in each ear, separately for L1 and L2). We included 18 trials with word pairs in the dichotic listening test. Words were presented simultaneously to both ears, spoken by a female speaker in neutral prosody. The time between each presented word pair was 800 ms, as suggested by an expert audiologist in the lab. Note that the dichotic listening test in our study was different from the one described by Westerhausen and Kompus (2018). These authors suggested that voluntary preference to selectively report one of the two stimuli, based on the instruction to attend to one ear, or triggered by arbitrary attentional strategies, should be considered when planning a dichotic-listening experiment. However, differences in difficulty between the two conditions might especially affect impaired individuals (as demonstrated in the clinical studies reviewed above), who may show a reduced number of correct left-ear identifications, and an inflated right-ear advantage. In short, we did not instruct our participants to focus on one ear. We used adobe audition for the simultaneous presentation of word pairs. Stimuli were carefully prepared without including any noise in the recorded voices.

### Procedure

The dichotic listening was completed in the same order for all participants. After obtaining written consent, participants received standardized instructions on the questionnaire and the task. The test was performed individually in a quiet room.

### Data Analysis

Data were analyzed using a repeated measures ANOVA in SPSS version 24. The ANOVA included the within-subject factors hemisphere (right and left), emotion (positive, negative, and neutral), and language (L1 and L2).

## Results

Mauchly’s test did not indicate any violation of the sphericity assumption for the ANOVA [Chi Square (2) = 1.325, *p* > 0.05]. The main effect of hemisphere (left/right) was significant [*F*_(1_,_59)_ = 92.689, *p* < 0.0001]. A significant main effect of language (L1/L2) [*F*_(1_,_59)_ = 419.162, *p* < 0.0001] indicated that the overall numbers of produced words in the first language was significantly higher than in the second language. A significant main effect of emotion was also observed [*F*_(1_,_59)_ = 29.451, *p* < 0.0001]. We further found significant two-way interactions between hemisphere and emotion [*F*_(1_,_59)_ = 11.684, *p* < 0.0001]; between hemisphere and language [*F*_(1_,_59)_ = 58.067, *p* < 0.0001]; and between emotion and language [*F*_(1_,_59)_ = 10.360, *p* < 0.0001]. Finally, the interaction of hemisphere, emotion, and language was significant [*F*_(1_,_59)_ = 4.880, *p* = 0.009]. Bonferroni-corrected *post hoc* paired *t*-tests (threshold: *p* < 0.004) showed a right ear advantage (left hemispheric advantage) for all words in L1 and for positive words in L2. In contrast, we observed a left visual field advantage (right hemispheric advantage) in L1 and L2 processing, regardless of emotion (see Table 1 and below for details).

**TABLE 1:** Results of *Post-hoc t*-test.

Pairs	Mean	Std. Deviation	95% confidence interval of the difference (lower)	95% confidence interval of the difference (upper)	*t*	Signature (two-tailed)
**Pair 1** **RApeL1 - LApeL1**	−2.30	1.58	−2.70	−0.1.90	−11.30	**0.001**
**Pair 2** **RAneL1 - LAneL1**	−1.23	1.68	−0.1.70	−0.80	−5.68	**0.001**
**Pair 3** **RAnuL1 -LAnuL1**	−2.28	1.67	−0.2.71	−1.85	−10.60	**0.001**
**Pair 4** **RApeL2 - LApeL2**	−1.03	1.54	−0.1.43	−0.64	−5.20	**0.001**
**Pair 5** **RAneL2 - LAneL2**	−0.52	1.70	−0.95	−0.80	2.37	0.021
**Pair 6** **RAnuL2 -LAnuL2**	0.57	1.51	−0.96	−0.18	−0.2.90	0.005

*Pair 1, right and left auditory positive words in first language; Pair 2, right and left auditory negative words in first language; Pair 3, RAnuL1 – LAnuL1: Right and Left Auditory Neutral words in first language; Pair 4, right and left auditory positive words in second language; Pair 5, right and left auditory negative words in second language; Pair 6, right and left auditory neutral words in second language, Bold font: survives a Bonferroni-correction for multiple comparisons (p < 0.004).*

As Table 1 shows, all but two differences between pairs of conditions were significant after Bonferroni correction. Results indicated a significant preference for the right ear relative to the left ear in L1 for positive words [*t*(59) = −11.30, *p* = 0.001]; negative words [*t*(59) = −5.68, *p* = 0.001]; and neutral words [*t*(59) = −10.60, *p* = 0.001]. Accordingly, for L2, a significant right ear preference was found for positive words [*t*(59) = −5.19, *p* = 0.001], but not for negative words [*t*(59) = −2.90, *p* = 0.021, does not survive the Bonferroni correction] or neutral words [(59) = −2.90, *p* = 0.005, does not survive the Bonferroni correction].

**FIGURE 1:**
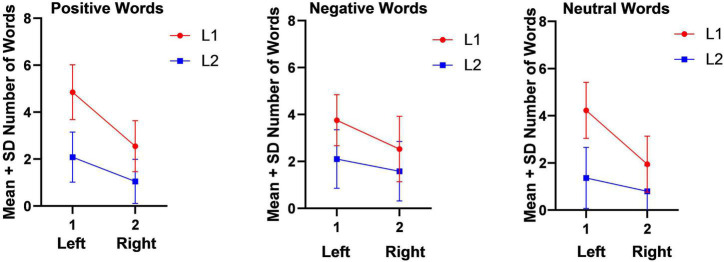
Mean and Standard deviation of Number of words in Dichotic Listening Task (Auditory Stimuli).

As Figure 1 shows, the number of emotional (positive and negative) and non-emotional (neutral) in L1 words which were heard and repeated back by the participants in the right ear (left hemisphere) were more than the repeated words in left ear (right hemisphere) in emotional (positive and negative) and non-emotional (neutral) words of L2. Figure 1 appears in sequence and is not copied from any other sources but just GraphPad Prism 8 software.

## Discussion

In this study, we examined hemispheric asymmetries in auditory processing of emotional and non-emotional words in L1 and L2. As a main finding, we observed a significant interaction effect of hemisphere, emotion, and language on the number of correctly repeated words. The right ear showed an advantage in the perception of emotional and non-emotional words in L1 and L2, except for neutral and negative words in L2.

Our first finding, that is, the advantage of the right ear (associated with the left hemisphere) in the processing of words independent of word type in the first language and positive words in the second language (and, as a trend, also for neutral and negative words in L2) is largely in line with previous work that showed left-hemispheric dominance for auditory gestures (Prieur et al., 2017). In that study, the Rennes laterality questionnaire was used to assess laterality for manipulation and communication. Our results extend these previous findings because we provide evidence that this effect may be independent of the emotion of a given word and does not strongly depend on the language (L1 or L2, although the effect was relatively stronger for L1). According to our results, at least positive emotional words in a bilingual’s second language (L2) are not different from emotional words in the first language (L1). The non-significant trend for negative and neutral words is most likely explained by the overall low power resulting from the low number of trials in our experiment. In a previous study, it was demonstrated that variations of bilingual processing of positive and negative information did not differ between L1 and L2 (Ong et al., 2017), but the processing of emotional words may be influenced by language proficiency and language complexity, which is perceived differently by participants. While our results indicated no difference in the processing of positive words of L1 and L2, a previous study reported higher perceived emotionality (i.e., valence and arousal) of words in L2 when presented in English compared to Chinese (Ong et al., 2017). In our study, the overall number of processed words was higher in L1 than L2 in the dichotic listening task, independent of word type, indicating a processing advantage for L1 which contrasts with a study in Chinese-English bilinguals that revealed an increased advantage just in processing of the positive words in L1 (Chen et al., 2015). In that study, responses to positive words were faster relative to negative words. Moreover, emotional words had higher accuracies than neutral words. The influence of word type, valence and exposure on the processing of emotional content was supported by the results from another previous study that included free recall and rating tasks from three groups of Arabic-English bilinguals (El-Dakhs and Altarriba, 2019). Specifically, these previous findings showed significant differences between emotion-label vs. emotion-laden vs. neutral words and negative vs. positive emotion words, and participants with increased L2 exposure generally outperformed those with less exposure.

Another study examined how the number of translations that characterize a word influences Spanish-English bilingual lexical organization and the processing of concrete, abstract, and emotional stimuli (Basnight-Brown and Altarriba, 2016). Concreteness effects emerged in both directions for words with more than one translation, which was explained by the bilingual memory representation. A strong right ear advantage was observed when words were presented in neutral prosody, which was decreased for all emotions (including furiousness, happiness, sadness, and fearfulness). There was no difference between ears as a function of perception of valence and discreetness of emotional words, demonstrating that all emotions had a similar effect in dichotic listening (Godfrey and Grimshaw, 2015). Other work shows that bilinguals are also faster than monolinguals in completing an ecologically valid object-finding task, indicating that they could ignore visual distractors and focus their attention on the relevant object better than monolinguals (Chabal et al., 2015).

We included a demographic questionnaire in our study, in which participants were asked questions about their first and second languages, such as the following examples: “At what age did you start learning English? How many percent of your daily conversations is in English? How did you learn the English language? What is your English test score and in which type of test? If you consider a native person’s level of English from 1 to 10, how do you rate yourself?” Based on the results from this questionnaire, we can assume that experience levels between L1 and L2 were not matched, but participants had more experience with their first language. This likely explains why our participants produced overall more words in L1. A previous study on the effects of bilingual proficiency on recognition memory in Spanish-English bilinguals supports our claim, demonstrating higher hit rates, better discrimination, and faster response times in the dominant language (Francis and Gutiérrez, 2012). These results support the idea that memory performance in the non-dominant language is impacted by both the greater demand for cognitive resources and the lower familiarity of the words. However, in our study, we did not assess verbal memory. Other studies observed reduced levels of vocabulary performance for bilinguals compared to monolinguals when lexical retrieval was measured under time constraints (Gollan et al., 2002; Ivanova and Costa, 2008). In line with these observations, another study suggested that relative to monolinguals, bilinguals may have slower access to words in their dominant language, but do not differ in terms of task accuracy if sufficient response time is allowed (Kaushanskaya et al., 2011). Moreover, bilingual lexical performance is highly sensitive to the bilinguals’ linguistic background. Performance shifts with increases in language proficiency have been documented for bilinguals’ lexical performance (Kroll and Stewart, 1994; Treffers-Daller, 2009) and bilinguals’ working memory skills (Service et al., 2002). These results show that overall task performance is strongly influenced by bilinguals’ language experience and history. Since bilinguals in our study were dominant in Persian, their performance was better in recalling words in L1 than L2.

Finally, the observed general differences of the auditory in word processing in our study should be considered. Left-hemispheric specialization of species-specific vocalizations in the auditory domain may have an evolutionary origin in non-primate mammals (Ehret, 1987), paralleling that in birds (George et al., 2005). Furthermore, it is well known that processing of human speech is primarily a function of the auditory areas of the left hemisphere while processing of tonal or melodic stimuli may be more readily accomplished by the right hemisphere. There is substantial evidence that the temporal/spectral acoustic properties of the stimulus, rather than the linguistic properties dictate the lateralization of processing (Zatorre and Belin, 2001; Schönwiesner et al., 2005). In general, auditory stimuli that are broad-band, rapidly changing or temporally complex, including speech and noise signals, including the words in our study, are preferentially processed in auditory areas of the left hemisphere (Zatorre et al., 2002; Tervaniemi and Hugdahl, 2003). As implicated by Sininger and Bhatara (2012), the left and right ears demonstrate asymmetric function reflecting the expected hemispheric asymmetry, and the asymmetry is based on the type of stimulus being processed, indicating right ear advantage for non-tonal stimuli.

### Limitations

Some limitations of our study need to be emphasized. First, the overall number of trials per condition was relatively low (*n* = 18) and should be increased in future studies to provide robust and reliable results. Secondly, the large number of within-subject variables may have influenced the results. Furthermore, our findings are limited to healthy young adults and may not be generalized to other populations.

## Conclusion

In summary, our findings provide new insight into processing advantages of both hemispheres for different word types. Overall, our results generally support previous studies reporting that late learning bilinguals show left-hemispheric dominance for auditory word processing. The observed differences in emotional word processing between L1 and L2 should be further investigated in future studies with larger stimulus sets and different collectives.

## Data Availability Statement

The original contributions presented in the study are included in the article/Supplementary Material, further inquiries can be directed to the corresponding author.

## Ethics Statement

The studies involving human participants were reviewed and approved by VN, Department of Psychology, Shahid Beheshti University, Tehran, Iran. The patients/participants provided their written informed consent to participate in this study.

## Author Contributions

RH conducted literature searches, provided summaries of previous research studies, conducted the statistical analysis under supervision of VN and GH, and wrote the first and finalized draft of the whole manuscript. VN designed the study. SA performed the data collection. GH revised the manuscript. All authors contributed to and have approved the final manuscript.

## Conflict of Interest

The authors declare that the research was conducted in the absence of any commercial or financial relationships that could be construed as a potential conflict of interest.

## Publisher’s Note

All claims expressed in this article are solely those of the authors and do not necessarily represent those of their affiliated organizations, or those of the publisher, the editors and the reviewers. Any product that may be evaluated in this article, or claim that may be made by its manufacturer, is not guaranteed or endorsed by the publisher.
