# Supplementary material for: Laterality in Emotional Language Processing in First and Second Language
Source: Front Psychol. 2022 Feb 3;12:736359. doi: 10.3389/fpsyg.2021.736359 (PMC8850280; doi:10.3389/fpsyg.2021.736359)
Supplement: Supplementary file 1 [file Presentation_1.pdf]

Supplementary Table 1. Presentation of Auditory word pairs in dichotic listening test to ears

Right Hemisphere	Left Hemisphere	Auditory Pairs	
		Left	Right
PEL2	PEL1	Love	لایق
NEL2	NEL1	Lost	ظالم
NUL2	NUL1	Key	کیف
PEL1	PEL2	مفید	Hope
NEL1	NEL2	فاجعه	Fever
NUL1	NUL2	چتر	Hat
NEL1	PEL1	گرفتار	بامحبت
NUL1	PEL1	پارچه	عاطفی
NUL1	NEL1	روسری	حادثه
PEL1	NEL1	صمیمی	مسخره
PEL1	NUL1	مشتاق	صندوق
NEL1	NUL1	جریمه	لیوان
NEL2	PEL2	Alone	Happy
NUL2	PEL2	Lamp	Nice
NUL2	NEL2	Paper	Dirty
PEL2	NEL2	Lucky	Loser
PEL2	NUL2	Wedding	Bottle
NEL2	NUL2	Patient	Column
PEL2	PEL1	Doll	عادل
NEL2	NEL1	Angry	گرسنه
NUL2	NUL1	Dress	تصویر
PEL1	PEL2	آراسته	Health
NEL1	NEL2	ناامید	Afraid
NUL1	NUL2	فرش	Air
NEL1	PEL1	بیمار	شکیبا
NUL1	PEL1	طناب	مودب
NUL1	NEL1	کلید	خاین
PEL1	NEL1	مهربان	بی-ارزش
PEL1	NUL1	بخشنده	پنجره
NEL1	NUL1	مقصر	کولر
NEL2	PEL2	Bored	Brave
NUL2	PEL2	Table	Honey
NUL2	NEL2	Clock	False
PEL2	NEL2	Music	Noisy
PEL2	NUL2	Beauty	Circle
NEL2	NUL2	Stress	Office

Supplementary Table 2. Mean and Standard Deviations of the words in SAM

Arousal Mean (SD)	Valence Mean (SD)	Words
3.52 (1.17)	1.57 (0.74)	جریمه
4.13 (1.24)	4.58 (1.01)	شادی
2.45 (1.40)	3.27 (0.95)	تصویر
2.98 (1.17)	1.87 (0.72)	گرسنه
1.47 (0.99)	2.90 (0.47)	فهرست
2.93 (1.42)	1.63 (0.71)	ناتوان
2.97 (1.44)	4.18 (0.94)	آراسته
3.07 (1.23)	1.42 (0.56)	ناامید
1.97 (1.24)	3.17 (0.94)	فرش
3.17 (1.39)	3.75 (1.56)	دوستانه
3.75 (1.05)	1.93 (0.89)	انتظار
1.00 (0.55)	2.70 (0.86)	لاستیک
2.27 (1.32)	3.82 (1.03)	شکیبا
3.33 (1.28)	1.42 (0.67)	بیمار
1.68 (1.20)	2.92 (0.59)	طناب
3.50 (1.44)	4.50 (0.77)	شوخ طبع
2.55 (1.30)	1.38 (0.64)	پژمرده
1.97 (1.13)	2.92 (1.09)	شمعدان
2.48 (1.14)	3.92 (1.13)	مودب
1.73 (1.23)	3.08 (0.76)	کلید
3.18 (1.42)	4.25 (0.83)	سخت کوش
3.60 (1.34)	1.33 (0.68)	خائن
1.43 (0.78)	2.87 (0.62)	کابینت
2.90 (1.45)	1.32 (0.59)	افسرده
2.28 (1.47)	3.28 (0.99)	پنجره
2.67 (1.14)	1.60 (0.86)	بی ارزش
3.18 (1.42)	4.30 (0.78)	مهربان
3.50 (1.49)	4.57 (0.59)	بخشنده
1.57 (0.98)	3.07 (0.68)	حوله
3.32 (1.25)	1.60 (0.82)	دلتنگ
3.88 (1.19)	1.30 (0.64)	حیله گر
2.82 (1.34)	3.88 (1.45)	با وقار
1.88 (1.23)	3.13 (0.94)	کولر
2.60 (1.49)	1.88 (0.84)	مقصر
1.78 (1.15)	2.78 (1.07)	مداد
3.42 (1.56)	1.47 (0.72)	حسود

Arousal Mean (SD)	Valence Mean (SD)	Words
3.58 (1.45)	4.45 (0.83)	سود
3.48 (1.22)	4.30 (0.96)	لایق
3.27 (1.35)	1.32 (0.56)	ظالم
3.30 (1.67)	1.67 (0.79)	خشن
1.82 (1.12)	3.02 (0.94)	کیف
2.35 (1.23)	3.53 (1.17)	آینه
2.85 (1.56)	4.20 (1.03)	مفید
3.88 (1.55)	4.32 (1.49)	موفق
4.00 (1.19)	1.32 (0.70)	فاجعه
3.65 (1.30)	1.57 (0.81)	عصبی
1.85 (0.97)	3.07 (0.97)	چتر
1.33 (0.68)	2.93 (0.86)	خط کش
3.40 (1.46)	4.55 (0.69)	با محبت
3.10 (1.24)	1.42 (0.64)	گرفتار
3.62 (1.15)	4.33 (1.10)	خوشی
3.63 (1.27)	1.18 (0.53)	شکست
3.53 (1.26)	4.08 (1.18)	عاطفی
1.57 (1.07)	2.87 (0.83)	پارچه
3.78 (1.34)	1.52 (0.77)	حادثه
1.47 (0.67)	3.13 (0.34)	دماسنج
3.57 (1.29)	4.15 (1.33)	تعطیلات
1.78 (1.16)	2.73 (0.91)	روسری
3.55 (0.92)	1.55 (0.91)	گناهکار
2.02 (1.35)	3.28 (0.76)	پاکت نامه
2.52 (1.17)	2.45 (1.09)	مسخره
1.58 (1.03)	2.95 (0.67)	صندوق
3.58 (1.53)	4.37 (0.80)	مشتاق
1.87 (1.18)	2.83 (1.15)	پیراهن
3.17 (1.52)	4.38 (1.01)	صمیمی
2.68 (1.25)	1.98 (1.04)	تنها
1.62 (1.09)	3.15 (0.48)	لیوان
2.23 (1.18)	4.03 (0.58)	آرام
3.68 (1.28)	4.22 (1.39)	بانشاط
1.50 (0.81)	2.93 (0.82)	دستگاه
2.82 (1.64)	1.55 (0.87)	چاپلوس
3.20 (1.28)	4.55 (0.81)	عادل

Supplementary Table 3. Power analysis and its outcome using G*Power software version 3.1.9.7

Input parameters	
Test family	F tests
Statistical test	ANOVA: Repeated measures; within factors
Type of power analysis	A priori: Compute required sample size, given alpha, power and effect size
Effect size	0.4
Alpha error probe	0.05
Power (1-beta error probe)	0.95
Number of groups	1
Number of measurements	3
Correlation among repeated measures	0.5
Non-sphericity correlation	1
Output parameters	
Non-centrality parameter Landa	17.28
Critical F	3.27
Numerator df	2
Denominator df	34
Minimum total sample size	18
**Actual Power**	**0.95**
